# Transient neuromyopathy after bromide intoxication in a dog with idiopathic epilepsy

**DOI:** 10.1186/2046-0481-65-19

**Published:** 2012-12-05

**Authors:** Sonja Steinmetz, Andrea Tipold, Thomas Bilzer, Henning Christian Schenk

**Affiliations:** 1 Department of Small Animal Medicine and Surgery, University of Veterinary Medicine Hannover, Bünteweg 9, Hannover, 30559, Germany; 2 Institute of Neuropathology, University of Düsseldorf, Moorenstrasse 5, Duesseldorf, 40225, Germany

**Keywords:** Epilepsy, Intoxication, Lower motor neuron signs, Myopathy, Potassium bromide

## Abstract

A seven-year old Australian Shepherd, suffering from idiopathic epilepsy under treatment with phenobarbitone and potassium bromide, was presented with generalised lower motor neuron signs. Electrophysiology and muscle-nerve biopsies revealed a neuromyopathy.

The serum bromide concentration was increased more than two-fold above the upper reference value.

Clinical signs disappeared after applying diuretics and reducing the potassium bromide dose rate. This is the first case report describing electrophysiological and histopathological findings associated with bromide induced lower motor neuron dysfunction in a dog.

## Background

In veterinary medicine, potassium bromide (KBr) is recommended as the drug of choice for treating recurrent generalised tonic-clonic seizures in dogs with hepatic insufficiency [1-3] and is frequently used as add-on therapy [3,4]. Potassium bromide is eliminated by the kidneys [5,6]. KBr monitoring in dogs is difficult because KBr has a long elimination half life of 25 to 46 days [7,8]. Side effects like general lethargy, general weakness, decreased mentation, ataxia, paraparesis, polyphagia and gastrointestinal affections may occur [9-14].

In dogs, also pancreatitis [15] and latency shifts in brainstem auditory evoked response are described [16].

To the authors’ knowledge, this is the first report describing a histopathologically confirmed peripheral neuromuscular alteration as a suspected consequence of bromide intoxication.

### Case presentation

A 7-year-old male Australian Shepherd, neutered, weighing 42 kg, treated with anti-epileptic drugs due to idiopathic epilepsy, was presented with a four-week history of weakness in the pelvic limbs, progressing to the thoracic limbs since a few days. The last seizure episode occurred 10 months previously. Idiopathic epilepsy had been diagnosed four years prior to this event by a complete neurological assessment [normal interictal neurological examination, complete blood cell count and serum biochemistry, magnetic resonance imaging (MRI^a^) of the brain and cisternal cerebrospinal fluid (CSF) analysis]. Anti-epileptic treatment was initiated with an initial dose of phenobarbitone 2.4 mg/kg BID PO (Luminal; Desitin). Due to inadequate seizure control KBr was added (10 mg/kg BID PO) by the referring veterinary surgeon. After 10 weeks a serum bromide concentration of 16.48 μmol/l was measured (VetMedLabor^b^, reference range 6–40 μmol/l). Three months later the KBr dosage was altered to 25 mg/kg BID PO by the referring veterinary surgeon attempting better seizure control. On several occasions, the owners further increased the dosage of both phenobarbitone and potassium bromide without contacting a veterinary surgeon. Four years after the initial consultation, the dog was presented at our institution for clinical signs of generalized gait abnormalities. On presentation in our clinic the dog received 4.9 mg/kg BID PO phenobarbitone and 101.19 mg/kg SID PO KBr for almost one year.

General physical examination revealed a severely obese dog (42 kg). Due to adiposity it was not possible to palpate the abdomen thoroughly. The rest of the general examination was unremarkable. Neurological examination revealed obtunded mental status, marked ambulatory tetraparesis, impaired postural reactions in all limbs, reduced spinal reflexes (extensor carpi radialis reflex, patellar reflex, tibialis cranialis reflex and flexor-withdrawal-reflex) and a bilateral decreased menace response. Neuroanatomic localisation was suggestive of a generalised lesion of the lower motor neuron system. Forebrain involvement could not be ruled out. Suspected etiological differentials included a metabolic-toxic (e.g. hypothyroidism), neoplastic (e.g. paraneoplastic syndrome, insulinoma), inflammatory and also a degenerative disease (e.g. storage diseases) [17,18]. Thoracic radiographs did not reveal any abnormality.

A CBC revealed a mild leucopenia (5.7 × 10^9^ leucocytes/l; reference range: 6.0-12.0x10^9^ leucocytes/l). Biochemistry profile indicated a mild increase in glutamate dehydrogenase (GLDH) 6.5 IU/l (reference range: 0–6 IU/l) and alkaline phosphatase (ALP) 962 IU/l (reference range: < 150 IU/l). Potassium and ionised calcium were within the reference range. Thyroxine (T4) and canine thyroid stimulating hormone (cTSH) were within normal limits (VetMedLabor^b^; T4: 34.75 nmol/l, reference 19–58 nmol/l; cTSH: 0.08 ng/ml, reference < 0.5 ng/ml). The serum bromide concentration was 45 mmol/l (ALOMED^e^, reference range: 8.75-18.75 mmol/l) and the serum phenobarbitone concentration was 168.52 μmol/l (VetMedLabor^b^, reference range: 43–172 μmol/l).

Anaesthesia was induced with 0.2 mg/kg IV levomethadone (L-Polamivet, Veterinaria AG), 0.5 mg/kg IV diazepam (Diazepam; Ratiopharm) and 1.2 mg/kg IV propofol (Narcofol; CP-Pharma) and maintained with isoflorane (Isofluran CP; CP-Pharma) in oxygen (Draeger respirator^c^). MRI^a^ of the brain and cisternal CSF-analysis were within normal limits, as previously diagnosed in 2005.

**Figure 1:**
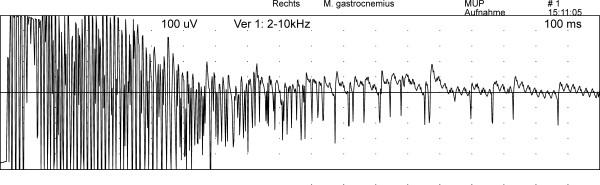
Myotonic discharges of the right gastrocnemius muscle.

Electrodiagnostic examinations (Nicolet Viking Quest IV^d^) were performed according to standard protocols of our neurophysiological laboratory [19]. One side electromyography of the appendicular, epaxial and masticatory muscles revealed abnormal spontaneous activity consisting of mild to moderate occurrence of positive sharp waves and fibrillation potentials in all examined muscles. In the gastrocnemius muscle these abnormal EMG patterns occurred more pronounced and together with occasionally recognised short episodes of myotonic discharges (700 ms in duration) (Figure 1). Due to this unexpected distribution pattern of abnormal activity, also the other side of the dog was examined and revealed a symmetrical electromyographic pattern. 

Motor nerve conduction velocity was measured in both peroneal nerves and in both radial nerves. It was impaired in the peroneal nerves of both pelvic limbs (mNCV: left: 52 m/s; right: 56 m/s [reference: 87.7 ± 6.8 m/s] [20]. Compared to the reference values in literature [21] the amplitudes in this patient were severely decreased (left distal stimulation 3.9 mV, left proximal stimulation 2.6 mV, right distal stimulation 6.2 mV, right proximal stimulation 3.7 mV). Furthermore the CMAP displayed a temporal dispersion with polyphasia. In both radial nerves the motor nerve conduction velocity was unremarkable.

Biopsies of the left gastrocnemius muscle and peroneal nerve were taken by standard procedures [22].

#### Histopathology

**Figure 2:**
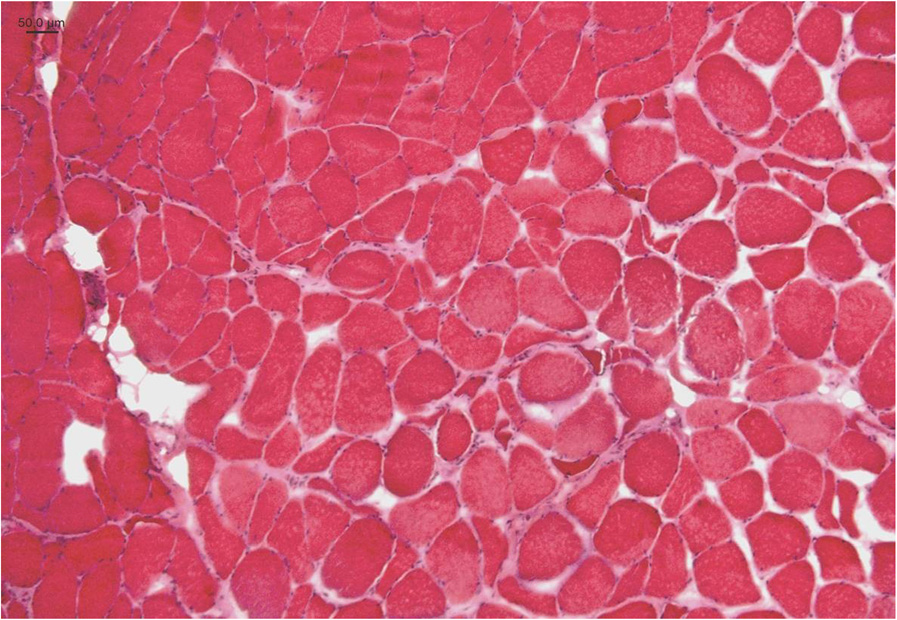
**M. gastrocnemius of the dog suffering from KBr intoxication: pathological variation of muscle fibre caliber sizes including neurogenic angular atrophies, moderate muscle fiber hypertrophies (according to the 50 μm scale in the upper left corner).** Endomysial fibrosis can be identified by the pink endomysial broadening especially in the lower part of the figure. Cross section, hematoxilin-eosin-staining.

**Figure 3:**
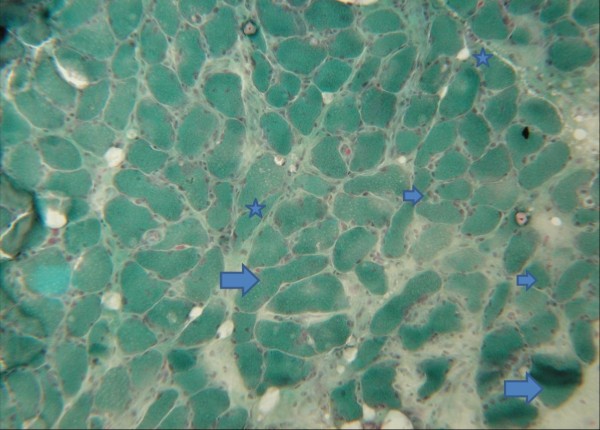
**Details of the M. gastrocnemius (Engel-stain): increase in the number of muscle cell nuclei and nuclear internalisation, endomysial fibrosis gives evidence for the chronicity of the process (lower right quarter).** Large arrows: fibre hypertrophies; small arrows: fibre atrophies; stars: fibres with a tendency to angular shape. No signs of mitochondriopathy producing red-stained rims in the muscle fibres periphery. Cross section, Gomori-trichrome-staining modified according to Engel; primary magnification: x50.

Cryostat sections of the native gastrocnemius muscle and the peroneal nerve were investigated after hematoxilin-eosin staining and Gomori-trichome-staining modified according to Engel, as well as NADH-TR/ATPase with pH of 4.6 and 9.4 for muscle fibre type differentiation and oil-red/black sudan and acidic phosphatase staining. Muscle and nerve tissue samples were frozen in isopentane at – 135°C and stored at −80°C until use. Readout of the muscle biopsy revealed a pathologically variable fibre type spectrum including moderate fibre atrophies as well as fibre hypertrophies. A moderate number of atrophic muscle fibres were of the angular type (Figures 2 and 3). Focal increase of myofibre nuclei, and of centrally located nuclei, as well as endomysial and perimysial fibrosis were also present. Muscle fibre type differentiation resulted in normal, atrophic and hypertrophic type 1 and type 2 fibres. There were no signs of inflammation and/or muscle fibre necrosis or marked mitochondrial alterations (Figure 3).

**Figure 4:**
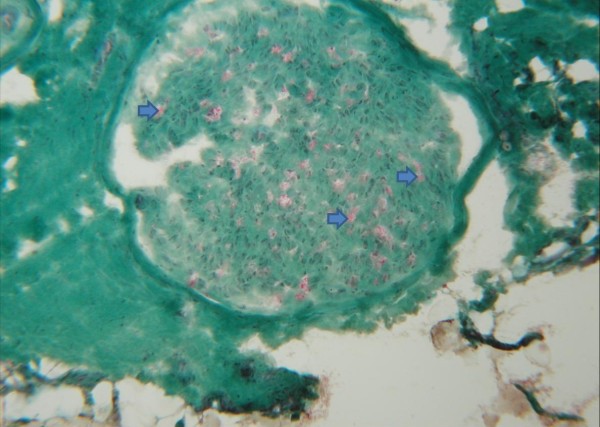
**Details of the N. peroneus of the same dog suffering from KBr intoxication.** Small peripheral nerve presenting only fragmentary myelination (arrows); variations of axonal diameters and loss of axons can be seen; endoneural fibrosis is evident by irregular green sectioning, as well as the increase of cell nuclei as a result of Schwann cell proliferation. Cross section, Gomori-trichrome-staining modified according to Engel; primary magnification: x50.

The peroneal nerve was examined under light microscopy. Structural changes were variable; in some of the investigated sections the relation of the axon to the myelin sheath appeared to be in a normal proportion to the axon-diameter, but mild individual axonal swelling and shrinking were seen. Other parts presented fragmentary / residual myelin, indicating a progressive demyelination (Figure 4). Endomysial fibrosis and Schwann cell proliferation were seen (Figure 4). Signs of active nerve regeneration or inflammation were absent. Blood vessels showed no pathological changes. These findings were compatible with a distinct chronic polyneuromyopathy of metabolic / or toxic etiology.

The dog was treated with intravenous administration of 0.9% sodium chloride solution (Isotonische Natriumchlorid-Lösung ad us. vet., B. Braun Melsungen AG), furosemide 1 mg/kg IV BID (Furosemid; Ratiopharm) and metamizol 25 mg/kg IV TID (Novalgin; Aventis Pharma). The antiepileptic drug dosages were reduced (phenobarbitone: initial dosage: 4.9 mg/kg BID PO, reduced dosage: 2.38 mg/kg BID PO, potassium bromide: initial dosage: 101,19 mg/kg SID PO, reduced dosage: 10.12 mg/kg SID PO). The dog was discharged after 9 days with mild gait abnormalities and an improved neurological state. Lower motor neuron signs disappeared completely seven days after being discharged. The serum phenobarbitone and serum bromide concentrations were controlled four weeks after discharge and were within normal limits (ALOMED^e^, phenobarbital 90 μmol/l, reference 65–130 μmol/l; bromide 12.39 mmol/l, reference 8.75-18.75 mmol/l). At the time of this report the dog experiences single generalized seizures every 4–5 months.

## Discussion

This case report describes a dog suffering from KBr intoxication after KBr treatment due to uncontrolled seizures. The dog presented generalized lower motor neuron signs. Further diagnostic investigations were consistent with a chronic neuromyopathy. This is the first time that neuromuscular signs due to bromide intoxication have been supported by electrophysiological and histopathological examinations.

In the present case axonopathy and myelinopathy were found. However, the primary target of bromide-intoxication could not be identified. The described patient was presented in a chronic state of the disease. At such a time point the initial damage (myelinopathy or axonopathy) cannot be determined. Moreover, bromide might affect directly muscle metabolism.

Finding the diagnosis for the presented case is challenging. As already described by Arezzo et al. (2011), electrophysiology and histopathology are excellent methods to describe toxic neuropathies, although each method has its limitation. Nerve biopsies have to be taken at specific locations, since histopathology may be unremarkable in proximal axons while axons in the distal segments may reveal distal axonopathy. Electrophysiology is a sensitive but not specific method. Changes such as reduced amplitudes in evoked responses may indicate structural and / or functional damage. In contrast minor structural damage can remain undetected by electrophysiological studies [23,24].

In the described case pathological spontaneous activity occurred in the muscles of all four limbs although marked EMG changes were particularly seen in both gastrocnemius muscles. The histopathological affection of both muscle fibre types indicates a neurogenic origin of the muscle lesion. A biopsy of the tibial nerve would have been beneficial, but due to the electrodiagnostic findings, the peroneal nerve has been chosen. The nerve biopsy revealed slight histopathological changes affecting axon and myelin not correlating well with the results of the mNCV measurements in the pelvic limbs. These contrasting findings raise the need for a more functional explanation of clinical signs. As bromide hyperpolarizes neuronal membranes by competing with chloride ions, the action potential generation and propagation is reduced [16]. Already March et al. (2002) suspected effects of bromide intoxication on auditory evoked potential (BAER) latencies. Significant latency shifts in waves I and V led to the assumption, that increased serum bromide concentrations delayed conduction along central and peripheral pathways. The current case report supports the hypothesis that bromide has a dose depending impact on the conduction in the peripheral nervous system. Lower motor neuron signs resolved when the bromide level declined. EMG abnormalities were detected in all examined muscles explaining the observed weakness in all four limbs in the absence of a reduced mNCV in the radial nerve. Spontaneous EMG activity seems to depend on disease duration and degree of distribution of pathologic mechanisms within the neuromuscular system [25]. The exclusion of other causes for the generalised lower motor neuron signs in the described dog can additionally support our theory.

Myotonic episodes observed in the gastrocnemius muscle might also be a sign of an altered chloride conduction induced by bromide intoxication. Myotonic potentials are often for example an electromyographic feature of myotonia congenita [26], a disease caused by a genetic mutation involving chloride channels. Excessive bromide levels could influence chloride conductance in the muscle membranes in the described dog leading to the clinical sign of weakness.

Peripheral neuropathy is a rare finding of methyl bromide intoxication in humans [27,28]. A dying-back axonopathy is suspected in this toxic event.

In veterinary medicine side effects of KBr treatment like ataxia, tremors, sedation, paresis, polyphagia, polydipsia and anorexia occurring in the initial weeks of treatment are frequently observed. Conscious proprioceptive deficits, hyporeflexia and anisocoria appear in some dogs with elevated serum bromide levels [29,30]. However, the described patient displayed lower motor neuron signs, including a marked ambulatory tetraparesis, hyporeflexia in the thoracic and pelvic limbs, as well as sedation and conscious proprioceptive deficits. Therapeutic drug monitoring and owner guidance are essential in order to avoid chronic KBr accumulation and severe side-effects [30]. In the study of Rossmeisl and Inzana (2009) dogs with potassium bromide intoxication had lower and upper motor neuron paresis, cranial nerve deficits, cerebellar ataxia and diffuse intracranial clinical signs [31]. These studies demonstrate that bromism can manifest as heterogeneous clinical signs of peripheral and central nervous dysfunction. In dogs morphological changes of the peripheral nervous system after bromide intoxication have not been examined and described. In this case report despite displaying histopathological changes of a neurogenic myopathy, the dog recovered completely after bromide dose reduction and facilitated renal excretion. A bromide induced neuromyopathy causing severe gait abnormalities and reduced spinal reflexes can be suspected as a presumed clinical diagnosis in the described dog. Other causes were ruled out and the response to treatment supports the diagnosis. Although phenobarbitone was reduced as well, the serum levels were always within the reference range and the dog never showed gait abnormalities before the add-on therapy with KBr was initiated. However, to have evidence-based proof that a bromide overdose was the only cause for lower motor neuron signs another electrodiagnostic work up and additional biopsies after clinical recovery should have been taken. Also re-inducing the clinical signs by increasing the dose of potassium bromide could haven proven the toxic event. The suspected diagnosis was based on history, laboratory findings and repeated neurological examinations.

Although some studies assume a dose-dependent effect [7,31,32] of bromide, clinical observations indicate that signs of bromide intoxication may be present independent of absolute serum bromide concentrations [16,33]. A possible explanation for these findings may be the duration of bromide therapy, slow or rapid dose increases, breed predispositions, concurrent central or peripheral nervous system diseases and the concurrent use of other anti-epileptic drugs [16,31].

## Conclusions

High doses of bromide may induce clinical signs of bromide intoxication due to chronic accumulation. Therefore, every six months blood parameters and bromide levels should be evaluated routinely to rule out any changes in serum level. Bromide intoxication may cause generalised lower motor neuron signs, EMG and mNCV abnormalities and muscle and nerve lesions which are consistent with a neuromyopathy.

## Footnotes

^a^ Magnetom Impact plus 1.0 Tesla, Siemens AG Medical Solutions Magnetiv Resonance Imaging, Forchheim, Germany.

^b^ VetMedLabor GmbH, Division of IDEXX Laboratories, Ludwigsburg, Germany.

^c^ Draeger respirator, Draeger Medical Techniques, Lübeck, Germany.

^d^ Nicolet Viking Quest IV, Nicolet EBE GmbH, Kleinostheim, Germany.

^e^ ALOMED, analytisches Labor Dr. Werner Müller, Radolfzell-Böhringen, Germany.

## Competing interests

None of the authors of this paper has a financial or personal relationship with other organizations or people that could influence or bias the content of the paper.

## Authors’ contributions

SS drafted the manuscript and helped to examine and manage the dog. AT helped to draft the manuscript. TB did the histopathological examination of the muscle and nerve biopsies. HCS did the neurological examination and the electrodiagnostics and supervised the management of the patient. All authors read and approved the final manuscript.
